# Metatarsal Pronation in Hallux Valgus Deformity: A Review

**DOI:** 10.5435/JAAOSGlobal-D-20-00091

**Published:** 2020-06-15

**Authors:** Emilio Wagner, Pablo Wagner

**Affiliations:** From the Departamento de Traumatologia, Clinica Alemana–Universidad del Desarrollo, Santiago, Chile.

## Abstract

Hallux valgus deformity is a multiplanar deformity, where the rotational component has been recognized over the past 5 to 10 years and given considerable importance. Years ago, a rounded shape of the lateral edge of the first metatarsal head was identified as an important factor to detect after surgery because a less rounded metatarsal head was associated to less recurrence. More recently, pronation of the metatarsal bone was identified as the cause for the rounded appearance of the metatarsal head, and therefore, supination stress was found to be useful to achieve a better correction of the deformity. Using CT scans, up to 87% of hallux valgus cases have been shown to present with a pronated metatarsal bone, which highlights the multiplanar nature of the deformity. This pronation explained the perceived shape of the metatarsal bone and the malposition of the medial sesamoid bone in radiological studies, which has been associated as one of the most important factors for recurrence after treatment. Treatment options are discussed briefly, including metatarsal osteotomies and tarsometatarsal arthrodesis.

Hallux valgus deformity has historically been defined as a varus deviation of the first metatarsal bone and a valgus orientation of the hallux, without considering additional components of the deformity. Classic teaching decades ago considered just the intermetatarsal angle to guide treatment, and certain algorithms were applied by this angular measure. Over the past 5 years, new information has been published regarding the multiplanar characteristic of hallux valgus deformity, and considerable importance has been given to the rotational deformity of the first ray.^1,2,3,4,5,6,7^

## Background

**Figure 1:**
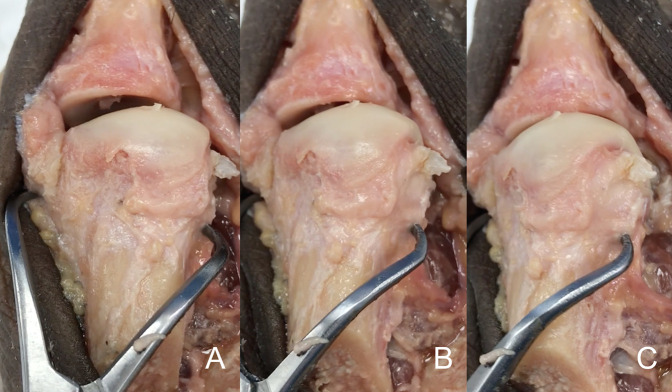
Photograph of the cadaveric specimen demonstrating the first metatarsal bone dissected free from soft tissues from dorsal aspect. A clamp is used to apply pronation to the metatarsal bone. **A**, The metatarsal bone in an anatomic position, without rotation applied. **B**, The same metatarsal bone with 15° of pronation applied to it. **C**, The same cadaveric bone with 30° of pronation applied. Note the change in the silhouette of the lateral aspect of the metatarsal head as the metatarsal condyle starts to appear in the view. As the pronation increases, the lateral aspect of the metatarsal head changes from a straight angle corner to a rounded corner, appearance given by the metatarsal condyle.

**Figure 2:**
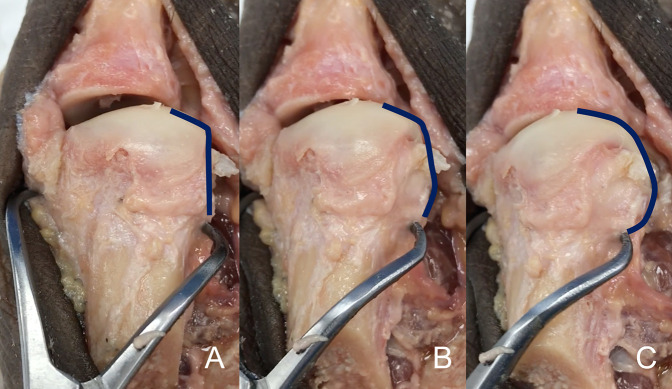
Photograph identical to the picture shown in Figure 1, with an inserted blue line which follows the lateral aspect of the metatarsal head to highlight the change in the contour of the bone, which will be perceived on radiographs as a lateral roundness. **A**, The metatarsal bone in an anatomic position, without rotation applied. **B**, The same metatarsal bone with 15° of pronation applied to it. **C**, The same cadaveric bone with 30° of pronation applied.

**Figure 3:**
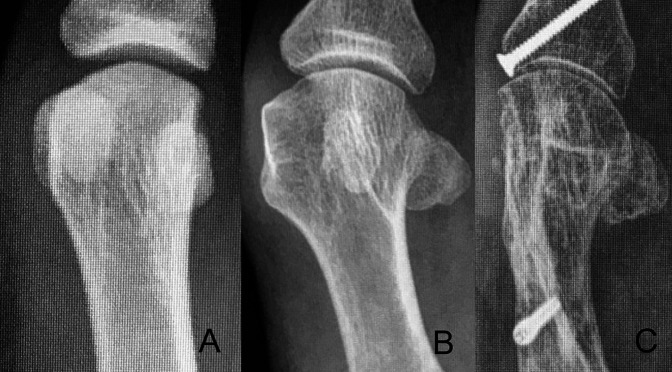
Photograph demonstrating the radiographic examples of first metatarsal bones with increasing pronation chosen to mimic the pronation shown in the cadaveric example of Figures 1 and 2.

The first reports found in the literature were published beginning in 1993 and used plain radiographs to describe the rotational deformity of the metatarsal, using morphological landmarks of the base of the first metatarsal to estimate rotation^8^ or using tangential weight-bearing radiographs.^9^ Almost no new research can be found in the literature dealing with metatarsal rotation, until 10 years later when Okuda et al^10^ published how the shape of the lateral aspect of the metatarsal head on AP radiographs of the foot became less rounded after hallux valgus surgery, which helped to decrease recurrence. The same author realized years later^11^ that supination of the metatarsal bone corrected this abnormal head shape, and thus concluded that pronation was involved in the deformity. An increased understanding of the relationship between an apparent first metatarsal round head shape and pronation was achieved after Yamaguchi et al^7^ published his work using digitally reconstructed radiographs from CT scans, demonstrating how rotation of the metatarsal was correlated with a round head shape(Figures 1–3).

**Figure 4:**
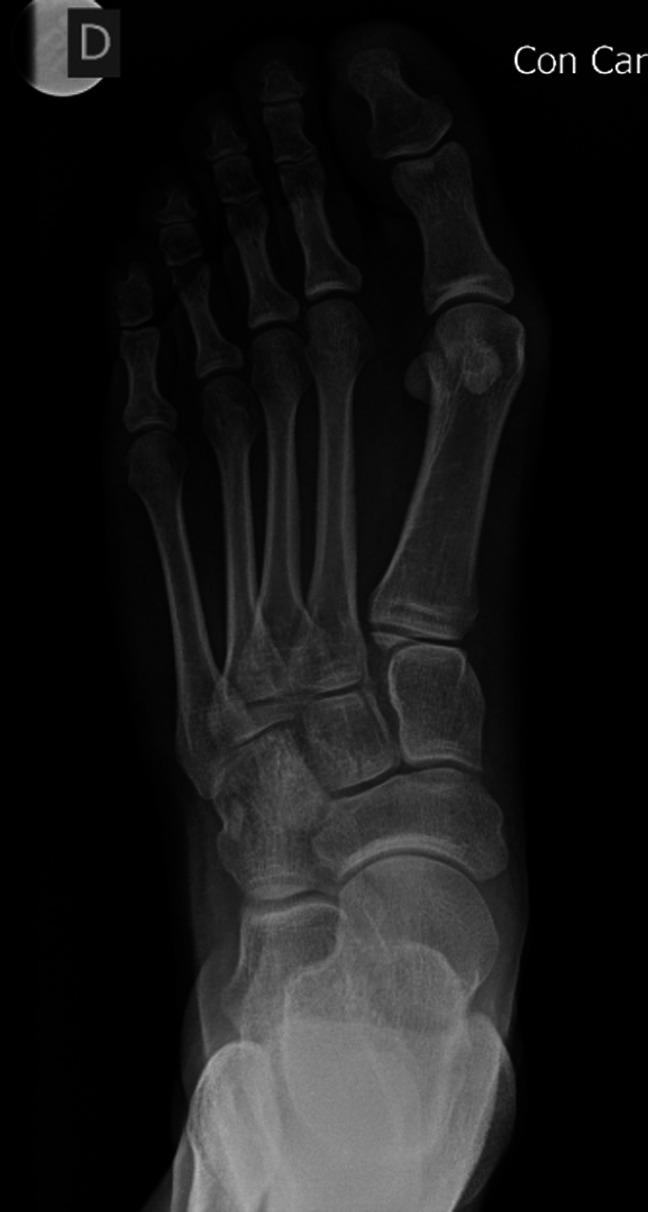
Radiograph demonstrating the anterioposterior section of a patient with hallux valgus. Note the apparent lateral sesamoid subluxation in relation to the metatarsal head.

**Figure 5:**
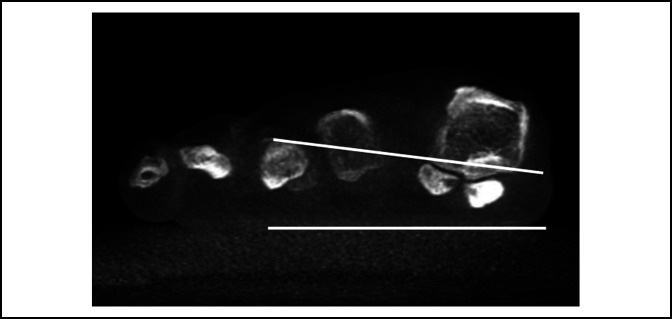
Photograph demonstrating the weight-bearing CT scan of the same patient. There is no subluxation of the lateral sesamoid. Two lines have been drawn to delineate the floor and the metatarsosesamoid facets, which demonstrate the pronation of the metatarsal bone. Owing to the pronation, when looking on the anterioposterior radiographic image, a seudosubluxation of the sesamoids is seen, and we start to perceive a roundness of the lateral aspect of the metatarsal head.

With the advent of simulated weight-bearing CT (WBCT) and cone beam WBCT, the concept of hallux valgus deformity as a multiplanar deformity became clearer. Four different scenarios have been shown to occur in patients with hallux valgus deformity relative to the relationship between the metatarsal head and the sesamoids, for example, hallux valgus with or without a pronated first metatarsal bone and with or without sesamoid subluxation.^2^ This implies that a group of hallux valgus patients exists in which the sesamoids seem to be subluxated on plain AP foot radiographs, but on CT, it is observed that a normal metatarsosesamoid relation exists and a pronated metatarsal explains the pseudosesamoid subluxation (Figures 4 and 5). In this same study, up to 87% of patients with hallux valgus deformity presented a pronated metatarsal, with and without sesamoid subluxation (61% and 26%, respectively). The values of metatarsal pronation reported in the literature vary, depending on how it is measured or estimated. Considering estimates taken from weight-bearing foot x-rays observing the first metatarsal head lateral edge roundness^7^ and the author's experience, the pronation can vary between 10° and 30°. Measurements taken from the first metatarsal axial view show pronation values between 4° and 14°,^12^ and if computerized geometric models are considered taken from simulated WBCT scans of the foot, the metatarsal rotation can be as high as 27° in patients with hallux valgus deformity.^13^ A study using partial WBCT scan has shown up to 22° of pronation in hallux valgus cases.^2^ Newer studies have shown that partial weight-bearing increases the pronation deformity in patients with hallux valgus deformity,^14^ and full weight-bearing increases the pronation of the first ray even in patients without hallux valgus deformity^15^ that highlights the importance of evaluating this deformity component.

## Biomechanical Consequences

Given the multiple ligamentous attachments (ie, medial capsule and deep intermetatarsal and metatarsosesamoid ligaments) between the first metatarsal bone and the hallux, when a hallux valgus deformity initially develops, the sesamoid complex rotates after the metatarsal, with or without dislocation. In long-standing deformities, the intermetatarsal ligament and adductor tendon keep their attachments to the lateral sesamoid, which together with a loose medial capsule and a constant lateralizing vector pull of the flexor hallucis longus tendon contribute to produce a dislocation of the sesamoids from the metatarsal sesamoid facets.^16–18^ Although no proven biomechanical consequences were observed for incongruent metatarsosesamoid joints, it has been described that as hallux valgus angle increases, so does the prevalence of cartilage lesions both in the metatarsophalangeal and in the metatarsosesamoid compartments.^19^ Therefore, it can be inferred that a well-aligned metatarsophalangeal joint will have a better biomechanical behavior and hopefully less deterioration over time.

The exact origin of the rotational deformity of the metatarsal bone is not known yet because the current available information only measures the first metatarsal bone rotation relative to the ground or to the second metatarsal, without evaluating whether the navicular or middle cuneiform bones also contribute to the deformity. It has been mentioned that pronation occurs at the cuneometatarsal joint but without scientific evidence,^3^ and more recently measurements taken with WBCT have shown that the pronation can be similar between patients with flatfeet and hallux valgus deformity, which suggests that the entire medial column participates in the final pronation of the metatarsal bone.^20^

The importance of identifying pronation deformity in a patient with hallux valgus deformity relies on our need to achieve a full correction of the deformity, on how to decrease the rate of recurrence, and how to hopefully prevent or stop metatarsosesamoid arthritis.

In 2012, it was suggested that our capacity to achieve an adequate correction was influenced by derotating the first metatarsal,^3^ and Okuda et al realized in his study that a supination stress of the great toe corrected the hallux valgus deformity, therefore suggesting that correcting pronation would help to achieve a correction of the deformity. Later studies dealing with tarsometatarsal arthrodesis have confirmed the importance of correcting metatarsal pronation because the first metatarsal became aligned in a Lapidus fusion only after supinating it (average 22° of supination).^1^ In this study, a strong statistical association was found between the tibial sesamoid position and the amount of supination required for joint alignment, which highlights the relationship between the sesamoid complex and the rotational deformity.

If we analyze the rate of recurrence after hallux valgus surgery, it has been reported to range from 2% up to 50%, depending on the population studied and on the definition of recurrence.^21^ Many factors have been associated with recurrence, such as postoperative sesamoid reduction,^22^ joint incongruity,^23^ preoperative greater hallux valgus angle, metatarsus adductus, and immediate postoperative hallux valgus angle of ≥8°.^24^ One of the latest studies published analyzing the factors associated to loss of correction in hallux valgus surgery, after adjusting for covariates, showed that only the tibial sesamoid position was positively associated. The tibial sesamoid position according to Hardy and Clapham represents the sesamoid subluxation, and it is determined by the position of the medial sesamoid relative to the centerline of the metatarsophalangeal joint on weight-bearing images, where 1 represents a normal position and 7 represents complete luxation. In the mentioned study,^25^ a tibial station greater than 4 was associated with a recurrence rate of 51%, using a very strict criteria of recurrence. Looking back at studies analyzing the relationship between tibial sesamoid position and coronal rotation of the first metatarsal, it has been shown that in 86% of the cases the tibial sesamoid position specifically depends on the coronal rotation of the metatarsal, thus allowing us to suggest that the coronal rotation is the main factor associated with recurrence.^2,16^

Finally, it has been shown that the radiographically perceived roundness of the first metatarsal head lateral edge is associated not only to a pronated metatarsal bone but also to a higher prevalence of metatarsosesamoid arthritis.^12^ Although it is not a level 1 study, it can be assumed that abnormal mechanics of the metatarsophalangeal joint because of its malalignment can lead to early deterioration of the cartilage (a common consequence for any misaligned joint), and thus, a correct alignment should be beneficial in stopping and/or preventing such damage.

## Diagnosis and Physical Examination

**Figure 6:**
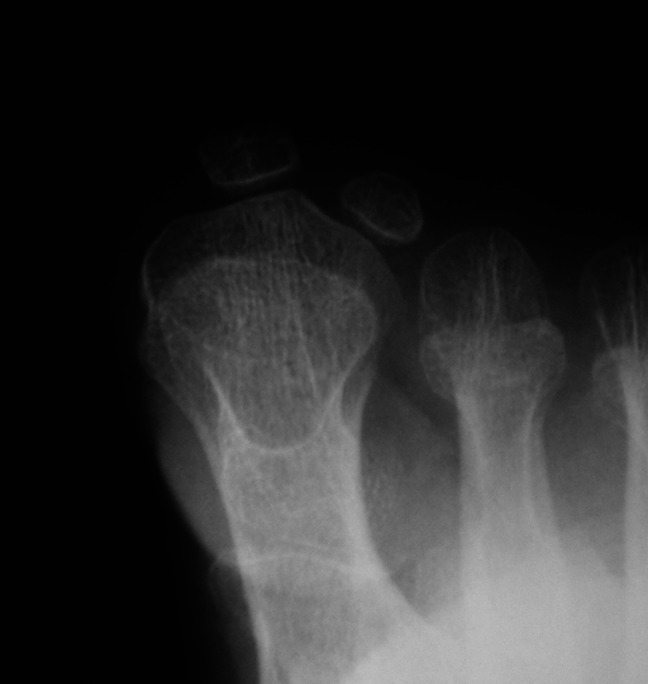
Photograph demonstrating the axial sesamoid view, where the difficulty to evaluate pronation is highlighted by the fact of an incomplete view of the foot and not a complete weight-bearing status.

**Figure 7:**
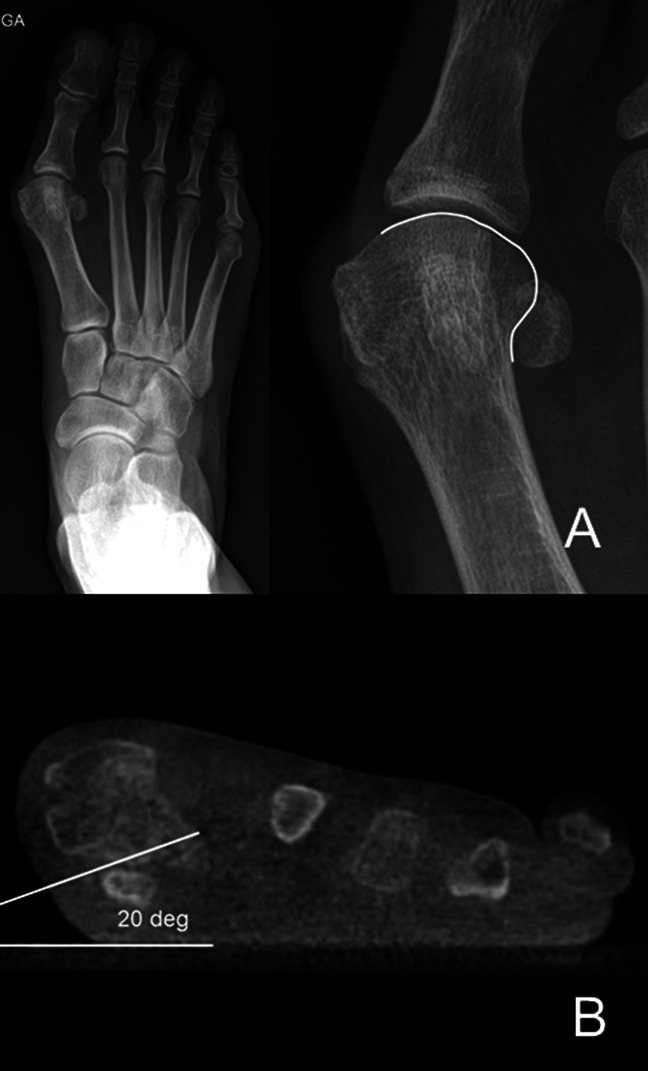
**A**, AP view of a hallux valgus case, where a magnification of the first metatarsal bone is on the right side of the image. The joint line is marked with a white line. If the joint line is followed onto the lateral aspect of the metatarsal head, it will represent the lateral metatarsal condyle, which gives the appearance of a rounded head. As in this case, the line is not “broken” but it is not possible to superimpose a perfect circle around the edge of the metatarsal; the predicted pronation is moderate (in the author's classification). **B**, Weight-bearing CT scan of the same patient presented in (**A**), where the measured pronation is 20**°**, between the horizontal weight bearing surface of the floor and the metatarso-sesamoid facets of the first metatarsal bone, confirming in this case a moderate pronation measurement (in the author's classification).

**Figure 8:**
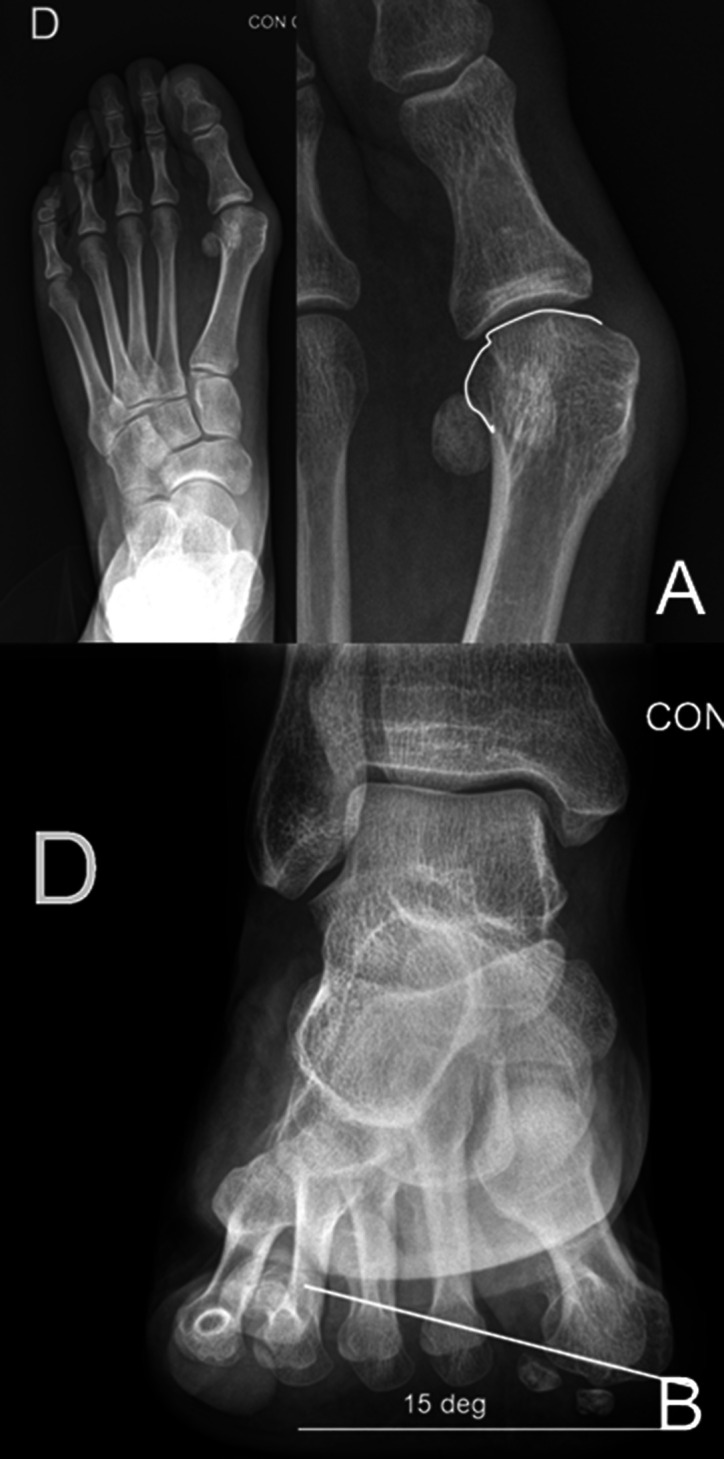
**A**, AP view of a hallux valgus case, where a magnification of the first metatarsal bone is on the right side of the image. The joint line is marked with a white line. If the joint line is followed onto the lateral aspect of the metatarsal head, it represents the lateral metatarsal condyle, which gives the appearance of a rounded head. As in this case, the line is “broken”; the predicted pronation is mild (in the author's classification). **B**, Bernard view of the same patient presented in (**A**). The pronation value of the first metatarsal bone is shown, measuring 15°, confirming in this case a mild pronation measurement (in the author's classification).

Patients presenting with hallux valgus deformity should be evaluated sitting and standing to evaluate the general alignment of the leg, ruling out hip, knee, or tibial deformities that may influence on the final alignment of the foot. Hindfoot alignment should also be evaluated because valgus or varus deviation will influence forefoot load and if symptomatic can be considered in the surgical plan. Bilateral standing evaluation is paramount to be able to evaluate the medial deviation of the first metatarsal and the final pronation of the hallux. Radiological examination is paramount to estimate metatarsal coronal deviation. The following four different methods exist described to estimate metatarsal rotation: using an axial sesamoid weight-bearing view, the Bernard view, an AP foot radiograph, and finally, a WBCT scan (Figures 4–8). It has been shown that the axial sesamoid view is not reliable because the sesamoids reduce back in position, regardless of their subluxated position on weight-bearing because of a bowstringing effect created by the flexor hallucis brevis with hallux dorsiflexion, which pushes the sesamoid complex under the metatarsosesamoid facets^2^ (Figure 6). The Bernard view suffers from the same unreliability because the image has to be taken with the first metatarsal axis perpendicular to the radiograph cassette, which most of the times is not possible, and the authors have seen in their practice that this view is erratic in its use. Relative to the WBCT scan, once available in most surgical centers, it should be the examination of choice because it can measure reliably the metatarsal rotation, besides the rest of the parameters generally measured in hallux valgus deformity, under weight-bearing conditions.^14,15^

**Figure 9:**
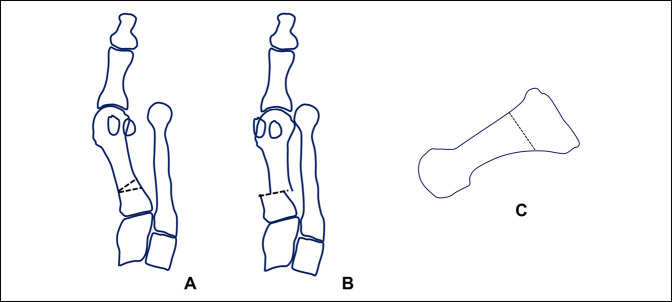
Diagram of the POSCOW osteotomy. **A**, Diagram represents the osteotomy marked on the metatarsal bone, consisting in a proximal lateral closing wedge transverse osteotomy with a lateral displacement. The pronation correction is performed without preoperative planning, rotating the distal fragment as needed intraoperatively. **B**, Diagram represents the correction already performed. **C**, Diagram shows the level of the osteotomy and its vertical orientation.

**Figure 10:**
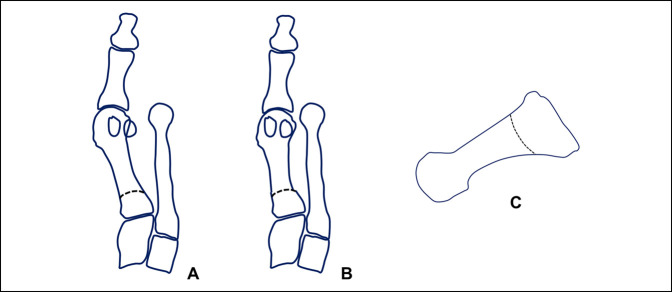
Diagram of the proximal supinating dome osteotomy. **A**, Diagram represents the osteotomy marked on the metatarsal bone, consisting in a proximal crescentic osteotomy. The pronation correction is performed without preoperative planning, rotating the distal fragment as needed intraoperatively. **B**, Diagram represents the correction already performed. **C**, Diagram shows the level of the osteotomy and its vertical orientation.

The last method used to estimate metatarsal rotation is by using the AP foot radiograph. As shown by Yamaguchi et al,^7^ a round shape on the lateral border of the metatarsal head is evidence of pronation given that the metatarsal condyles appear on an AP view as the metatarsal externally rotates. This method has been used by the authors, where a classification of pronation in stages has been suggested to make it easier to plan and decide on a specific osteotomy to use.^26,27^ To use this method, the metatarsophalangeal joint line must be delineated and continued onto the lateral aspect of the metatarsal head. On the lateral aspect, the metatarsal head silhouette is formed by the lateral metatarsal condyle, and the more the metatarsal pronation increases, the better the metatarsal condyle is seen, therefore providing a more rounded metatarsal head on AP foot x-rays (Figures 1–3). We have shown (personal data, in consideration for publication) that the roundness of the lateral aspect of the metatarsal head correlates with the amount of pronation the metatarsal head presents. In this way, if the lateral aspect of the metatarsal head is completely round and a circle can be superimposed onto it, the estimated pronation angle is 30° or more. If the lateral contour of the metatarsal head is rounded without any “step” but not as round as a circle, the estimated pronation angle is 20° to 30° (Figure 7, A and B). If the metatarsal head presents any step on its lateral aspect, the estimated pronation angle is 10° to 20° (Figure 8, A and B). Finally, if a straight angle is formed between the metatarsophalangeal joint line and the lateral aspect of the metatarsal head, there is no pronation at all.

## Treatment Options

Few surgical options exist designed to correct metatarsal pronation because most of the options generally use displacement osteotomies as the chevron or the scarf, which can correct the metatarsus varus deviation, but not any rotation. To the best of our knowledge, the procedures capable of correcting pronation of the metatarsal are osteotomies such as the proximal oblique sliding closing wedge osteotomy,^28^ the proximal metatarsal dome osteotomy,^29^ or the proximal rotational metatarsal osteotomy^27^ and fusions, such as the Lapidus procedure^30^ or the first metatarsophalangeal fusion. We will explain briefly some characteristics of them in the next paragraph.

**Figure 11:**
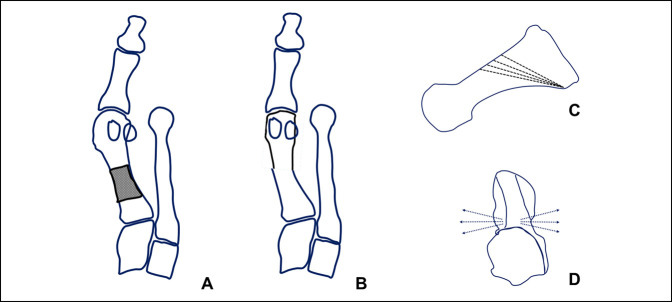
Diagram of the PROMO osteotomy. **A**, Diagram represents the osteotomy marked on the metatarsal bone, consisting in an oblique distal dorsal to plantar proximal osteotomy, where correction is achieved rotating the distal fragment along the osteotomy plane, achieving correction of both the varus and the pronation deformity (**B**). Preoperative planning is performed, which determines multiple possible osteotomy orientations in the sagittal and axial planes (**C** and **D** correspondingly) being able to accurately correct any combination of metatarsal varus and pronation.

**Figure 12:**
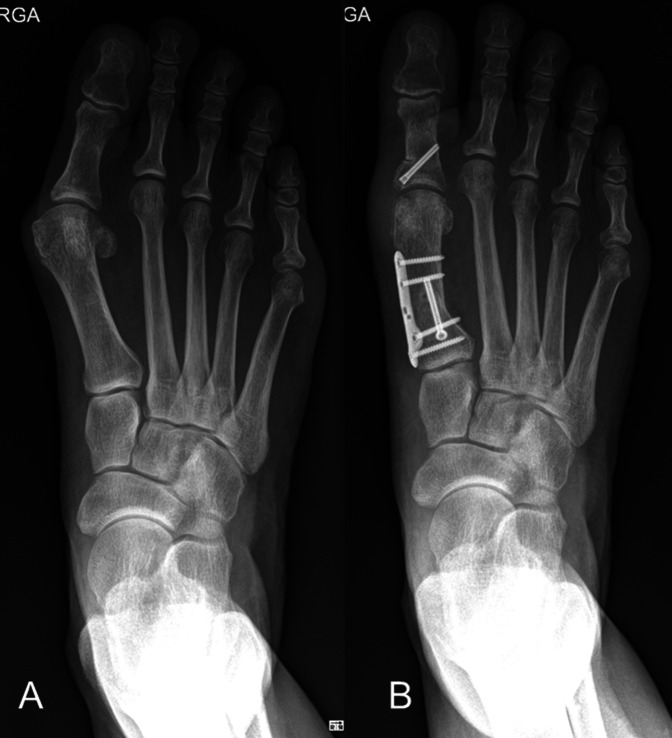
Preoperative and postoperative example of hallux valgus case treated with a PROMO osteotomy of the first metatarsal and an akin osteotomy of the proximal phalanx of the hallux.

The proximal oblique sliding closing wedge osteotomy is a proximal oblique slide closing wedge osteotomy done on a transverse plane, and it can derotate the metatarsal and thus correct the metatarsal pronation (Figure 9). Owing to this, it is very powerful but unstable osteotomy, needing a lot of care when doing it and when loading it. The same care and concern exist with the proximal supination osteotomy or dome osteotomy (Figure 10). The Lapidus arthrodesis has been performed lately also correcting the pronation of the metatarsal^1,30^ with very good results. The drawback of the Lapidus fusion is the fact of the required shortening of the first ray and the fusion itself, with certain known biomechanical consequences such as increased plantar pressure beneath the first ray (in case of first ray depression), second and third metatarsal (in case of first ray elevation), and increased joint pressure in the naviculocuneiform joint and fifth metatarsocuboid joint.^31^ These previous factors raise concern when indicating this procedure specially in younger patients with high physical activity. Finally, the proximal rotational metatarsal osteotomy^27^ relies on specific cutting jigs and different plane angulations of a proximal oblique osteotomy starting dorsal distal and ending proximal plantar, being able to correct both transverse and coronal plane deviations of the metatarsal through its design (Figures 11 and 12). Very good results have been published with this technique too.

## Summary

Hallux valgus recurrence is the most frequent complication in hallux valgus surgery. No consensus exists about how to define recurrence, and therefore, varying degrees of relapse rate are mentioned in the literature, being as low as 16% or as high as 50%.^21^ The factors involved in hallux valgus recurrence have been identified in the immediate postoperative period and include an immediate hallux valgus angle greater than 8°, postoperative sesamoid position of grade 4 or greater, metatarsal pronation, metatarsus adductus, and severe hallux valgus preoperatively.^24^ If we use an adequate surgical correction technique with adequate correction power,^32^ we will improve the correction of the varus of the metatarsal and the valgus of the big toe, but we will not necessarily improve the correction of the sesamoid position. This undercorrection has been cited as the main cause of recurrence in several other studies,^33,34^ and as stated in the first paragraphs of this article, tibial sesamoid position is related directly to the pronation of the metatarsal.^2^ Therefore, we can suggest that correcting the metatarsal pronation is of utmost importance specially if we want to improve our tibial sesamoid position correction postoperatively, and techniques capable of achieving the mentioned correction should be considered in our surgical armamentarium. More work has yet to be done to know exactly which are normal values of metatarsal pronation, how to measure it in a reliable and easy way, and how to correctly address the rotational deformity in patients with hallux valgus.
